# Effect of Electric Fields on the Mechanical Mechanism of Regorafenib–VEGFR2 Interaction to Enhance Inhibition of Hepatocellular Carcinoma

**DOI:** 10.3390/biom15010042

**Published:** 2025-01-01

**Authors:** Yichen Tian, Fenghui Liao, Heng Sun, Yongrong Lei, Yuna Fu, Feng Xia, Jianhua Wang

**Affiliations:** 1 Key Laboratory of Biorheological Science and Technology, Ministry of Education, College of Bioengineering, Chongqing University, Chongqing 400044, China; 201919021135@cqu.edu.cn (Y.T.); liaofenghui@stu.cqu.edu.cn (F.L.); sunheng@cqu.edu.cn (H.S.); 20161902085@cqu.edu.cn (Y.L.); fuyuna@cqu.edu.cn (Y.F.); 2 Key Laboratory of Hepatobiliary and Pancreatic Surgery, Institute of Hepatobiliary Surgery, Southwest Hospital, The First Hospital Affiliated to AMU (Southwest Hospital), Chongqing 400038, China

**Keywords:** mechanical mechanism, regorafenib, VEGFR2, electric fields, hepatocellular carcinoma, AFM

## Abstract

The interaction between molecular targeted therapy drugs and target proteins is crucial with regard to the drugs’ anti-tumor effects. Electric fields can change the structure of proteins, which determines the interaction between drugs and proteins. However, the regulation of the interaction between drugs and target proteins and the anti-tumor effects of electric fields have not been studied thoroughly. Here, we explored how electric fields enhance the inhibition of regorafenib with regard to the activity, invasion, and metastasis of hepatocellular carcinoma cells. We found that electric fields lead to an increase in the normal (adhesion) and transverse (friction) interaction forces between regorafenib and VEGFR2. In single molecule pair interactions, there are changes in specific and nonspecific forces. Hydrogen bonds, hydrophobic interactions, and van der Waals forces are the main influencing factors. Importantly, the increase in the adhesion force and friction force between regorafenib and VEGFR2 caused by electric fields is related to the activity and migration ability of hepatocellular carcinoma cells. The morphological changes in VEGFR2 prove that electric fields regulate protein conformation. Overall, our work proves the drug–protein mechanical mechanism by which electric fields enhance the anti-tumor effect of regorafenib and provides insights into the application of electric fields in clinical tumor treatment.

## 1. Introduction

Malignant tumors pose a significant threat to human life [1]. Currently, targeted therapy stands as one of the primary clinical treatments for tumors [2,3,4]. A range of inhibitors have been developed to target various proteins, including tyrosine kinase inhibitors (TKIs) [5]. The effective interaction between molecular targeted therapy drugs and target proteins is crucial to the anti-tumor effects of these drugs [6,7,8]. The anti-tumor effect of molecular targeted therapy drugs may be affected by weakened interaction caused by a reduced or absent ability to bind to the target protein [9,10].

It has been reported that electric fields can alter the physical and chemical properties and the structure of proteins [11,12,13]. The conformation of the protein determines the binding and interaction between the drug and the target protein. The lock-and-key relationship and appropriate structure of both the protein and the drug are decisive factors for the efficacy of the drug [14,15]. Changes in protein conformation can either enhance or inhibit drug efficacy [16]. In addition, tumor-treating fields (TTFields), as a non-invasive treatment that applies low-intensity, intermediate-frequency, alternating electric fields locally, has been applied clinically [17,18]. This raises the question of whether electric fields could be used as an adjuvant therapy to modify the conformation of target proteins in order to enhance drug–protein binding and improve the anti-tumor effect of targeted therapy.

Our previous studies have found that binding and interaction between drugs and proteins are accompanied by changes in the mechanical behavior of proteins. The interaction between proteins in a drug solution indirectly reflects the interaction between the drug and protein [19,20]. The binding of the drug to the protein obstructs the original binding between proteins, resulting in a decrease in the interaction force between the proteins. Atomic force microscopy (AFM) has shown great technical advantages in the field of protein–molecule interaction force measurement [21,22]. The molecular interaction mechanism of a protein complex can be understood by characterizing the intermolecular interaction with nanoscale single-molecule force spectroscopy [23].

Regorafenib is currently the preferred TKI for the treatment of advanced hepatocellular carcinoma (HCC); this drug inhibits cell proliferation and tumor malignant progression by inhibiting the phosphorylation of tyrosine residues of target proteins such as VEGFR2 [2,3]. In this study, we aimed to explore whether electric fields can enhance the inhibitory effect of regorafenib on HCC and to verify the correlation of this with enhanced regorafenib–VEGFR2 interaction forces. The results will provide a new direction for the application of electric fields in clinical cancer therapy.

## 2. Methods

### 2.1. Electric Field Stimulation

An in vitro electric stimulation device made in-house was used in this study (Supplementary Figure S1). A pair of stimulation electrodes were inserted through the cover of the cell culture dish and were perpendicular to the bottom of the dish. The diameter of the cell culture dish was 10 cm. The positive and negative electrodes of the stimulation electrode were both made of platinum and were kept parallel. The distance between these electrodes was 6.5 cm, and their size was 10 × 20 × 0.2 mm. The stimulation electrodes were connected to an external power supply, which was used to apply constant electric fields with intensities of 20, 50, and 100 mV/mm. Cells were subjected to electric stimulation after 12 h of drug treatment. When the cells were to be stimulated with an electric field, the device was placed in the cell incubator, and the electrodes were immersed in the culture medium for 1 h. The electrodes were immersed in the drug solution at room temperature, and the samples for AFM measurement were stimulated for 6 h.

### 2.2. Preparation and Characterization of Protein Molecular Layer

The MHA (16-mercaptohexadecanoic acid) (Sigma-Aldrich, Saint Louis, MO, USA) molecular layer was prepared by incubating the gold-coated substrates (Ted Pella, Redding, CA, USA) in 1 mM MHA solution for 24 h. The MHA molecular layer was incubated in a mixture of 2 mg/mL NHS (N-Hydroxysuccinimide) (Sigma-Aldrich, Saint Louis, MO, USA) and 2 mg/mL EDC (1-Ethyl-3-(3-dimethylaminopropyl) carbodiimide) (Sigma-Aldrich, Saint Louis, MO, USA) solutions for 1 h. Then, the MHA molecular layer was incubated in VEGFR2 (ABclonal, Wuhan, China) solution (1 mM) at 4 °C for 12 h to obtain VEGFR2 protein molecular layers. MHA, as a bridging molecule, forms a S-Au bond with the gold substrate at one end through its thiol group, while the carboxyl group at the other end is activated by EDC and NHS to form an amide bond with the lysine residues of the VEGFR2 protein, thereby linking the VEGFR2 protein to the substrate surface. The modification of the AFM probes (Budget Sensors, Sofia, Bulgaria) was as same as that of the gold-coated substrates. The morphology of the bare gold-coated substrate and VEGFR2 molecular layer was scanned by AFM [24,25]. The surface particle distribution, height, and particle size were analyzed by CSPM Imager software v4.7. The static contact angle of the gold-coated substrates was measured at room temperature (25 °C) in air using a contact angle meter (SDC-200S, Shengding Precision Instrument Co., Ltd., Dongguan, China). Ultrapure water was used as the probe solution.

### 2.3. AFM Measurement

AFM measurements were performed with a JPK NanoWizard II atomic force microscope (JPK Co., Berlin, Germany) under SMFS mode at 25 °C. Rectangular silicon cantilevers with overall gold coating (nominal spring constant of 0.2 N/m) and square pyramidal tips were employed for the force measurement. Bare silicon tips of the same specification were used for image collection.

The adhesion force and friction force measurements were performed in contact and FFM (friction force microscopy) modes, respectively. The scanning rate was 1.0 Hz, the scanning range was 2 × 2 μm^2^, and the resolution of the two-sided scanning was 512 × 512 pixel^2^. During the measurement, the tip distance was kept constant using closed-loop feedback. The AFM measurements were recorded as voltage–distance curves (V-d curves) and friction loop curve. According to Hooke’s law, F = kd, the V-d curves were converted to force–distance curves.

### 2.4. Cell Lines and Cell Culture

The human HCC cell lines LM3, HepG2, and Huh7 were purchased from the Cell Bank of Shanghai Institute of Cell Biology (Shanghai, China). These cell lines were identified through cell line authentication by STR (short tandem repeat) profiling. Cells were cultured in Dulbecco’s modified Eagle’s medium (DMEM) (Gibco, Grand Island, NY, USA) containing 10% fetal bovine serum (FBS) (Gibco, Grand Island, NY, USA) at 37 °C and 5% carbon dioxide. The cell lines used in the experiments were within 20 passages.

### 2.5. Cell Activity Assay

The CCK-8 (Cell Counting Kit-8) (Beyotime Biotechnology, Shanghai, China) was employed for the cytotoxicity assessment of HepG2 and Huh7 cells. A 1 × 10^4^ Huh7 or 5 × 10^3^ HepG2 cell suspension was seeded into each well of a 96-well plate and incubated for 24 h. A 5 μM solution of regorafenib (MCE, Monmouth Junction, NJ, USA) was added to the cells and incubated for 24 h. The medium was removed, and fresh medium (200 μL) was added to the cells. Then, 10 μL of CCK-8 solution was added to each well and incubated for another 2 h. The UV-Vis absorption was measured at 450 nm by Thermo Scientific Varioskan Flash (Thermo Scientific, Waltham, MA, USA).

### 2.6. Cell Proliferation Assay

Huh7 or HepG2 cells were cultured in DMEM containing regorafenib at a concentration of 5 μM for 12 h. Then, the cells were stimulated with a 0/20/50/100 mV/mm electric field for 1 h. The medium was removed at the end of the stimulation. The cells were trypsinized and resuspended in new medium. A 2 × 10^5^ Huh7 or HepG2 cell suspension was seeded into each well of a 24-well plate and incubated for 24 h. A 5 μM solution of regorafenib was added at the time of cell seeding. Cell proliferation was detected with the BeyoClick™ EdU Cell Proliferation Kit (Beyotime Biotechnology, Shanghai, China). Then, the cells were fixed with 500 μL of 4% formaldehyde for 20 min at room temperature. The cells were then washed with PBS three times. The cell nuclei were stained with 200 μL of DAPI (Beyotime Biotechnology, Shanghai, China) for 10 min. Fluorescence detection was performed with a fluorescence microscope (Olympus, Tokyo, Japan).

### 2.7. Protein Expression Assay

Western blotting was used to detect protein expression. Total protein was extracted from the lysate and heated at 100 °C for 10 min to denature it. The total protein was isolated by SDS-PAGE (Beyotime Biotechnology, Shanghai, China) and transferred to a 0.45 μm NC (nitrocellulose) membrane (Merck KGaA, Darmstadt, Germany). The NC membrane was blocked with 5% milk for 1 h and incubated with primary antibodies at 4 °C for 12 h. Specific primary antibodies included anti-P21 antibody (Abcam, Cambridge, UK), anti-P53 antibody (Abcam, Cambridge, UK), and anti-GAPDH antibody (Abcam, Cambridge, UK). Then, the NC membrane was incubated with goat anti-rabbit IgG heavy and light chains (Abcam, Cambridge, UK) at room temperature for 2 h to allow binding between the primary antibody and the secondary antibody. Immunoreactivity was detected using ECL (enhanced chemiluminescence) substrates (Thermo Scientific, Waltham, Massachusetts, USA) and recorded using a ChemiDoc imaging system (Bio-Rad, Hercules, CA, USA).

### 2.8. Immunofluorescence

LM3 cells were cultured for 12 h in DMEM containing regorafenib at a concentration of 5 μM. The cells were then stimulated with an electric field for 1 h. The medium was replaced at the end of the electric stimulation. The LM3 cells were trypsinized and resuspended in new medium. A total of 2 × 10^5^ LM3 cells were inoculated into 24-well plates with cover slides sterilized with 75% ethanol. After culturing for 24 h, the cells were fixed with 500 μL of 4% formaldehyde for 10 min at room temperature. The coverslips were then washed with PBS three times, and the cells were permeabilized with 0.03% Triton X-100. Nonspecific binding sites were blocked with a solution of 1% bovine serum albumin in PBS for 30 min. The cells were combined with primary antibodies for 24 h. The coverslips were then washed with PBS three times, and the cells were incubated in secondary antibodies for 2 h. The coverslips were then washed with PBS three times. The cell nuclei were stained with 200 μL of DAPI (Beyotime Biotechnology, Shanghai, China) for 10 min. Finally, the images were collected by fluorescence microscopy (Olympus, Tokyo, Japan) after sealing. The primary antibodies were anti-phospho-VEGFR2 (ABclonal, Wuhan, China), anti-phospho-FAK (ABclonal, Wuhan, China), anti-α-Smooth Muscle Actin (ABclonal, Wuhan, China), and anti-Snail (ABclonal, Wuhan, China). The secondary antibodies were Cy5-conjugated goat anti-rabbit IgG (H+L) (Abcam, Cambridge, UK) and Alexa Fluor^®^ 488-conjugated secondary antibody (Abcam, Cambridge, UK).

For cytoskeleton staining, 200 µL of Alexa Fluor 594 phalloidin (Solarbio, Beijing, China) was used to stain the cytoskeleton for 30 min after the cells were permeabilized with 0.03% Triton x-100. The coverslips were then washed with PBS three times. The cell nuclei were stained with 200 μL of DAPI (Beyotime Biotechnology, Shanghai, China) for 10 min. Finally, the images were collected by fluorescence microscopy (Olympus, Tokyo, Japan) after sealing.

### 2.9. Wound Healing Assay

LM3 cells were cultured for 12 h in DMEM containing regorafenib at a concentration of 5 μM. The cells were then stimulated with an electric field for 1 h. The medium was replaced at the end of the electric field stimulation. The LM3 cells were trypsinized and resuspended in new medium. LM3 cells were plated in a 6-well plate and grown to confluence. Then, the cell layers were scratched for the wound-healing assay. A linear wound was created by dragging a 10 μL pipette tip through the monolayer. The cells were washed with PBS. At 0 and 24 h after the scratch was made, the cell migration was observed and photographed by using an inverted microscope (Olympus, Tokyo, Japan). Image J software 1.52e (NIH, Bethesda, MD, USA) was used to measure the area of the linear wound.

### 2.10. Transwell Assay

LM3 cells were cultured for 12 h in DMEM containing regorafenib at a concentration of 5 μM. The cells were then stimulated with a 0/20/50/100 mV/mm electric field for 1 h. The medium was removed at the end of the stimulation. The LM3 cells were trypsinized and resuspended in new medium. A 24-well plate with 8 μm pore size polycarbonate membrane inserts (CORNING, Corning, NY, USA) was used to evaluate cell motility. For the migration assay, 5 × 10^4^ LM3 cells were seeded in the upper chamber in 300 μL of serum-free DMEM, and 800 μL of DMEM with 20% serum was added to the lower chamber. After culturing for 24 h for the migration assay, the non-migrated cells were removed from the upper chamber. The cells that had passed through the membrane were fixed with 4% formaldehyde and stained with 0.1% crystal violet. Five visual fields were randomly selected from each membrane, and the cell numbers were counted via a microscope (Olympus, Tokyo, Japan).

### 2.11. Young’s Modulus of Cells

The NanoWizard II atomic force microscope (JPK, Berlin, Germany) was used to measure the Young’s modulus of cells. The probe model employed was HQ: CSC38/Cr-Au-50 (MikroMasch, Watsonville, CA, USA). Huh7 or HepG2 cells were cultured for 12 h in DMEM containing regorafenib at a concentration of 5 μM. The cells were then stimulated with a 0/20/50/100 mV/mm electric field for 1 h. The medium was removed at the end of the stimulation. The cells were trypsinized and resuspended in new medium. A total of 5000 Huh7 or HepG2 cells were seeded onto a cell culture dish that had been treated with Matrigel. After incubating for 24 h, the culture medium was removed, and the dish was washed with PBS to eliminate non-adherent cells and impurities. The cells to be tested were observed and identified under an inverted microscope (Leica, Wetzlar, Germany). Ten points around the nucleus of the cells were selected for testing, with each point measured five times. All measurements were conducted in PBS solution. The measured force curves were analyzed via JPK SPM Data Processing software 3.4 (JPK Instruments AG, Berlin, Germany).

### 2.12. Statistical Analysis

The force data were summarized using Microsoft Excel 2021 (Microsoft, Redmond, WA, USA). Gaussian fitting of force data was performed using the nonlinear curve fitting module of OriginPro 2022 (OriginLab, Northampton, MA, USA). Statistical significance was calculated by performing unpaired t-tests using GraphPad Prism version 8.3.0 (GraphPad Software, La Jolla, CA, USA). Herein, “ns” is used to mean “not statistically significant”, while * *p* < 0.05, ** *p* < 0.01, *** *p* < 0.001, and **** *p* < 0.0001 denote statistically significant differences.

## 3. Results

### 3.1. Electric Fields Enhance the Inhibitory Effect of Regorafenib on the Proliferation of HCC Cells

**Figure 1:**
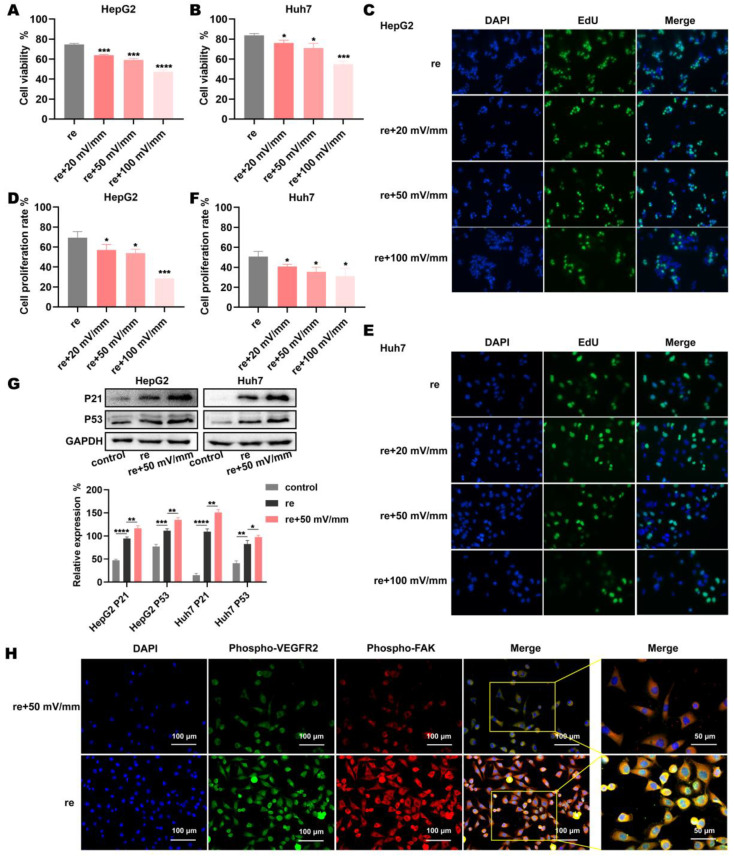
Electric fields enhance the inhibitory effect of regorafenib on the viability and proliferation of HCC cells. (**A**,**B**) The viability of HepG2 and Huh7 cells when using regorafenib alone and when using regorafenib in combination with electric fields. (**C**–**F**) The proliferation of HepG2 and Huh7 cells when using regorafenib alone and when using regorafenib in combination with electric fields. (**G**) Protein levels of P53 and P21 in HepG2 and Huh7 cells that received no treatment, regorafenib alone, and regorafenib in combination with electric fields, Western blot original images can be found in supplementary. (**H**) Immunofluorescence of phosphorylated VEGFR2 and phosphorylated FAK in LM3 cells after treatment with regorafenib alone and in combination with electric fields. * indicates *p* < 0.05; ** indicates *p* < 0.01; *** indicates *p* < 0.001; and **** indicates *p* < 0.0001.

In this study, the HCC cell lines Huh7 and HepG2 were used to conduct a drug sensitivity assay to verify whether electric fields enhance the anti-tumor effect of regorafenib at the cellular level. It was found that the electric fields had a damaging effect on HCC cell lines within the range of intensities set in this study (Supplementary Figure S2A). In order to exclude the effect of cytotoxic products produced by electric stimulation on cell activity, the medium was replaced immediately after the cells were electrically stimulated in this study. When Huh7 and HepG2 cells were treated with 5 μM regorafenib, the electric fields of 0 (control), 20, 50, and 100 mV/mm were applied. The results show that the latter three electric fields enhanced the inhibitory effect of regorafenib on the activity of Huh7 and HepG2 cells (Figure 1A,B). Compared with the control group, the proliferation of HCC cells was significantly reduced when the electric fields of 20, 50, and 100 mV/mm were applied (Figure 1C–F). With the increase in intensity of the electric field, the activity and proliferation of Huh7 cells and HepG2 cells decreased (Figure 1A–F). Compared with the control group, the protein expression levels of the tumor suppressors P53 and P21 were significantly increased under a 50 mV/mm electric field, suggesting that the proliferation of Huh7 and HepG2 cells treated with regorafenib was further inhibited by this electric field (Figure 1G and Figure S2B). In addition, the HCC cell line LM3 was selected for the immunofluorescence assay. It was found that compared with regorafenib treatment alone, the phosphorylation of VEGFR2 and its downstream signaling pathway FAK in HCC cells was decreased after treatment with 50 mV/mm electric fields and regorafenib (Figure 1H). Therefore, it can be hypothesized that the electric fields activated the inhibitory effect of regorafenib on the phosphorylation of the target protein VEGFR2, thereby enhancing the inhibition of HCC cell line proliferation. This may be related to the fact that the electric fields improved the drug efficacy of regorafenib.

### 3.2. Electric Fields Enhance the Inhibitory Effect of Regorafenib on Invasion and Metastasis by HCC Cells

**Figure 2:**
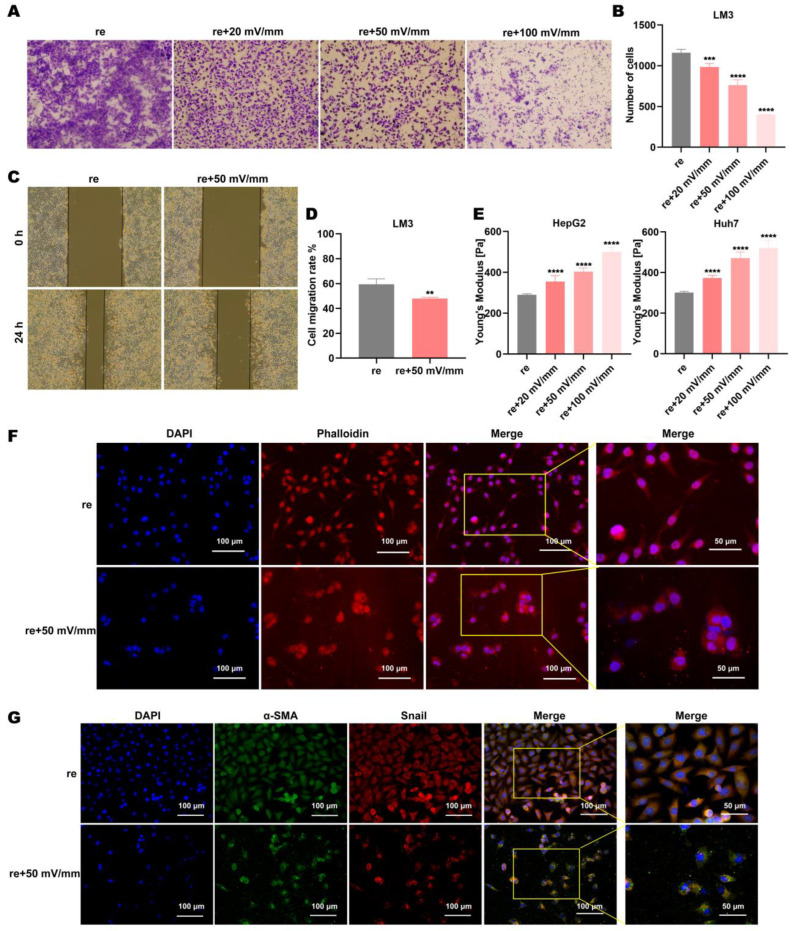
Electric fields enhance the inhibitory effect of regorafenib on invasion and metastasis by HCC cells. (**A**,**B**) Transwell assay of LM3 cells when using regorafenib alone and when using regorafenib in combination with electric fields. (**C**,**D**) Wound healing assay of LM3 cells when using regorafenib alone and when using regorafenib in combination with electric fields. (**E**) The Young’s modulus of HepG2 and Huh7 cells when using regorafenib alone and when using regorafenib in combination with electric fields. (**F**) The cytoskeleton of LM3 cells when using regorafenib alone and when using regorafenib in combination with electric fields. (**G**) Immunofluorescence of α-SMA and Snail in LM3 cells after treatment with regorafenib alone and in combination with electric fields. ** indicates *p* < 0.01; *** indicates *p* < 0.001; and **** indicates *p* < 0.0001.

Regorafenib acts on the target protein VEGFR2 to inhibit tumor angiogenesis and inhibit tumor metastasis [26,27,28]. Tumor invasion and metastasis are important indicators to evaluate the efficacy of drugs [29,30]. We selected the HCC cell line LM3 for cell migration assays. The wound healing assay and Transwell assay results showed that LM3 cells that underwent co-treatment with regorafenib and electric fields were less able to migrate than those treated with regorafenib alone (Figure 2A–D). With the increase in intensity, the migration ability of LM3 cells was significantly inhibited (Figure 2A–D). Young’s modulus represents the softness of the cell [31,32]. Previous studies have shown that softer cells, with lower a Young’s modulus, are more likely to be invasive and metastatic [33,34]. With the increase in the intensity of electric fields, the Young’s modulus of Huh7 and HepG2 cells gradually increased, that is, the flexibility of cells gradually decreased, and the invasion and metastasis ability of the cells gradually decreased (Figure 2E). The softness of cells is closely related to the cytoskeleton [35]. To further verify the effect of electric fields on the cytoskeleton, phalloidin was used to stain the cytoskeleton of LM3 cells (Figure 2F). When LM3 cells were treated with regorafenib alone, the cytoskeleton showed a spindle shape that was more prone to migration, and the cell pseudopod was clear (Figure 2F). However, when the LM3 cells were treated with regorafenib and the 50 mV/mm electric field, the cytoskeleton changed significantly, showing an oval shape with weak migration ability and the pseudopod of the cells disappeared (Figure 2F). Immunofluorescence results showed that compared with regorafenib alone, the expression of α-smooth muscle actin (α-SMA) and Snail in LM3 cells was decreased after treatment with the electric field and regorafenib (Figure 2G). Snail overexpression can induce the epithelial–mesenchymal transition, thereby improving cell invasion and migration ability [36,37]. High expression of α-SMA is associated with tumor metastasis [38]. Above all, these results suggest that electric fields can increase the inhibitory effect of regorafenib on invasion and metastasis by HCC cell lines and alter the cytoskeleton.

### 3.3. Electric Fields Affect the Adhesion Force Between Regorafenib and the Target Protein VEGFR2

**Figure 3:**
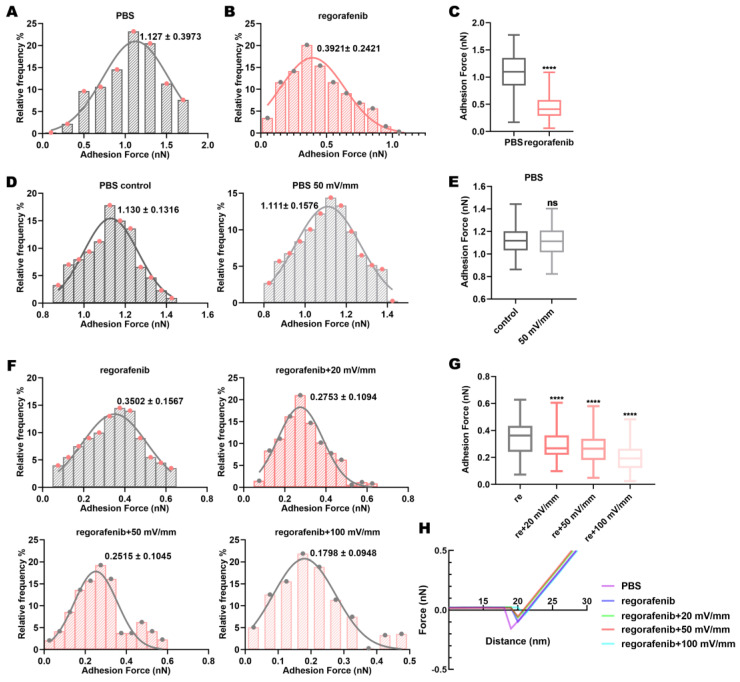
Electric fields reduce VEGFR2-VEGFR2 adhesion force in regorafenib solution. (**A**) Distribution of VEGFR2-VEGFR2 adhesion force in PBS and (**B**) regorafenib solutions; (**C**) comparison between these forces. (**D**) Distribution of VEGFR2-VEGFR2 adhesion force in PBS solution alone and in PBS solution combined with electric fields; (**E**) comparison between these forces. (**F**) Distribution of VEGFR2-VEGFR2 adhesion force in regorafenib solution alone and in regorafenib solution combined with electric fields; (**G**) comparison of these forces. (**H**) Force–distance curve. The values in the adhesion force distribution graph represent the mean ± SD values. “ns” indicates “not statistically significant”; and **** indicates *p* < 0.0001.

Electric fields can induce protein movement [13,39]. Drug–protein binding may be accompanied by changes in force and energy [19]. We examined VEGFR2-VEGFR2 adhesion force in regorafenib solution using AFM. Adhesion force is a kind of normal interaction force between molecules when they are relatively stationary [40]. The change in VEGFR2-VEGFR2 adhesion force in regorafenib solution is used to reflect the change in adhesion force between regorafenib and VEGFR2 (Supplementary Figure S2C). It was found that compared with the control group (PBS solution), the VEGFR2-VEGFR2 adhesion force in the regorafenib solution was significantly reduced (Figure 3A–C). This demonstrated the binding of regorafenib to VEGFR2 and the increasing adhesion force between regorafenib and VEGFR2. In addition, the applied 50 mV/mm electric field could not affect the VEGFR2-VEGFR2 adhesion force in PBS solution (Figure 3D,E). The effect of electric fields on the VEGFR2-VEGFR2 adhesion force was excluded. Importantly, compared with treatment using regorafenib solution alone, the VEGFR2-VEGFR2 adhesion force in regorafenib solution under electric fields of varied intensities was reduced (Figure 3F,G). That is, the adhesion force between regorafenib and VEGFR2 was enhanced. Moreover, the VEGFR2-VEGFR2 adhesion force in regorafenib solution decreased with the increase in intensity (Figure 3F,G). This implied that the interaction between regorafenib and VEGFR2 was enhanced as the electric field strength increased. The adhesion force data follow a Gaussian distribution (Figure 3F). Representative force curves are presented in Figure 3H. The changes in the interaction forces reflect the changes in the binding strength between the drug and the protein. The binding strength between a drug or ligand and a protein depends on the structure and properties of the protein. The energy provided by the electric fields may enable VEGFR2 to quickly overcome the energy barrier required for conformational transition, thereby increasing the probability of VEGFR2 binding to regorafenib. Furthermore, the conformational changes induced by the electric fields may cause changes in the hydrogen bonds, van der Waals forces, and hydrophobicity between VEGFR2 and regorafenib, thereby altering the adhesion force.

### 3.4. Electric Fields Affect the Specific and Non-Specific Forces Between Regorafenib and the Target Protein VEGFR2

The total adhesion force obtained from the force curves in AFM is the sum of multiple discrete bonds with a finite number of interacting molecules and within a fixed contact area. The specific interaction forces *F_i_* and non-specific interaction forces *F*_0_ within VEGFR2-VEGFR2 complexes can be decoupled using the following formula [19].

*σ*^2^*_m_* = *μ_m_F_i_* − *F_i_F*_0_ (1)

**Figure 4:**
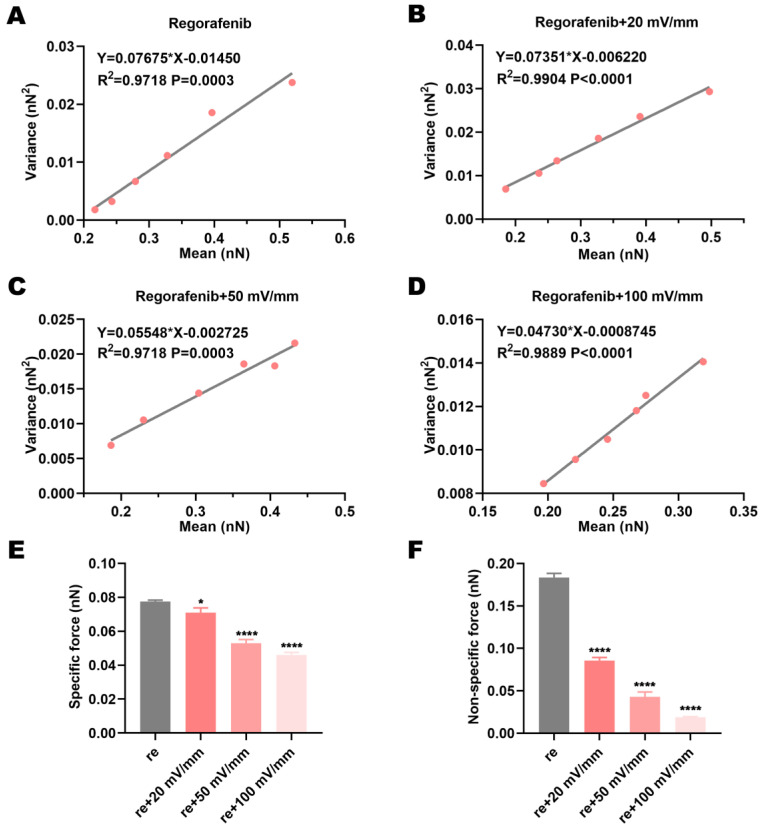
Electric fields reduce the specific and non-specific forces of VEGFR2-VEGFR2 in regorafenib solution. (**A**–**D**) Linear relationship between the mean and variance of adhesion force. (**E**) The specific and (**F**) non-specific VEGFR2-VEGFR2 forces in regorafenib solution alone and in regorafenib solution combined with electric fields. * indicates *p* < 0.05; and **** indicates *p* < 0.0001.

For our measurement, we can plot the average value (*µ_m_*) as the abscissa and the variance (*σ^2^_m_*) as the ordinate according to Equation (1), and then *F_i_* (corresponding to the slope) and *F*_0_ (corresponding to the intercept) of the VEGFR2-VEGFR2 complex can be obtained. With the mean value as the horizontal coordinate and the variance as the vertical coordinate, the plots revealed a good linear relationship between them (Figure 4A–D). From the slope and intercept, *F_i_* and *F*_0_ for VEGFR2-VEGFR2 complexes in regorafenib solution or combined with electric fields could be obtained (Figure 4A–D). Compared with treatment using regorafenib alone, the specific and non-specific forces of VEGFR2-VEGFR2 complexes in regorafenib solution under electric fields of varied intensities decreased (Figure 4E,F). Moreover, with the increase in intensity, the specific and non-specific varied intensity forces decreased (Figure 4E,F). The reduced specific force suggests that the VEGFR2-VEGFR2 hydrogen bonding interaction may decrease. By contrast, the reduced non-specific force suggested that the van der Waals force, electrostatic interaction, and hydrophobic interaction of VEGFR2-VEGFR2 complexes may decrease. The reduced specific and non-specific VEGFR2-VEGFR2 forces reflected the binding of regorafenib and VEGFR2. It was suggested that the specific and non-specific forces between VEGFR2 and regorafenib had corresponding changes. Moreover, the non-specific force of VEGFR2-VEGFR2 pairs decreased more sharply than the specific force under electric fields (Figure 4E,F). This means that the binding and interaction of regorafenib and VEGFR2 promoted by electric fields mainly interfered with the non-specific VEGFR2-VEGFR2 force.

### 3.5. Electric Fields Affect the Friction Force Between Regorafenib and the Target Protein VEGFR2

**Figure 5:**
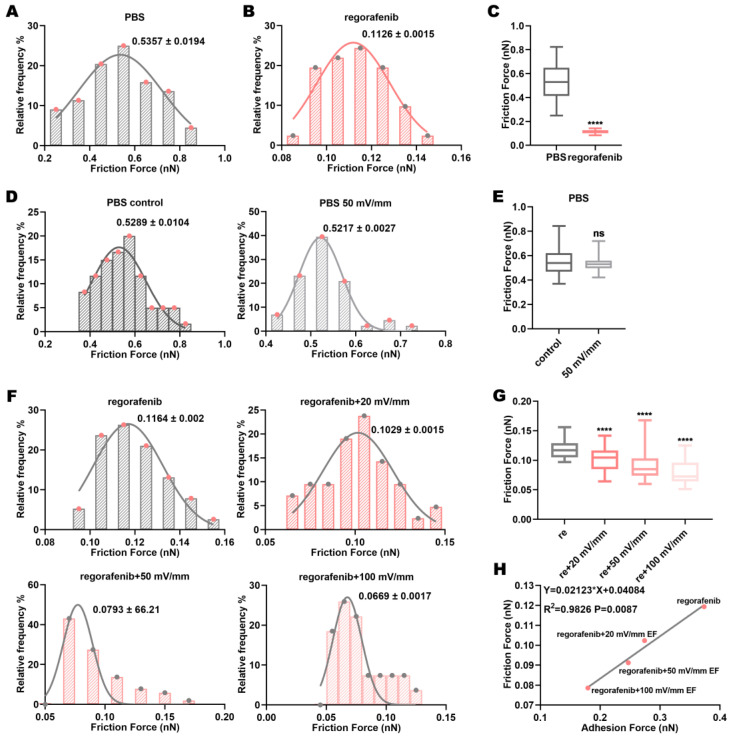
Electric fields reduce the VEGFR2-VEGFR2 friction force in regorafenib solution. (**A**) Distribution of VEGFR2-VEGFR2 friction force in PBS and (**B**) regorafenib solutions; (**C**) comparison of these forces. (**D**) Distribution of VEGFR2-VEGFR2 friction force in PBS solution alone and in PBS solution combined with electric fields; (**E**) comparison of these forces. (**F**) Distribution of VEGFR2-VEGFR2 friction force in regorafenib solution alone and in regorafenib solution combined with electric fields; (**G**) comparison of these forces. (**H**) The correlation between the friction force and adhesion force between VEGFR2 molecules after treatment with regorafenib alone or in combination with electric fields. The values in the friction force distribution graph represent the mean ± SD values. “ns” indicates “not statistically significant”; and **** indicates *p* < 0.0001.

Friction force is also a form of molecular interaction [41,42]. Friction force is the form of adhesion force in different pulling directions [43]. Considering the many possibilities of the angle of drug binding to proteins, the friction force should be considered to verify the changes in the interaction force between the drug and the protein. AFM was used to detect the friction force within VEGFR2-VEGFR2 pairs in the regorafenib solution. The change in the friction force within VEGFR2-VEGFR2 pairs in the regorafenib solution was used to reflect the change in the friction force between regorafenib and VEGFR2 (Supplementary Figure S2B). It was found that the VEGFR2-VEGFR2 friction force in the regorafenib solution was significantly lower than that in the control group (PBS solution) (Figure 5A–C). Regorafenib bound to VEGFR2 and reduced the contact surface between VEGFR2 and VEGFR2, thereby reducing the friction force between molecules of VEGFR2. This proved that the binding of drugs to proteins is multi-directional. The results showed that the 50 mV/mm electric field did not affect the VEGFR2-VEGFR2 friction force in PBS solution (Figure 5D,E). A possible effect of electric fields on the VEGFR2-VEGFR2 friction force was excluded. Importantly, compared to treatment with regorafenib alone, the VEGFR2-VEGFR2 friction force in regorafenib solution was reduced under electric fields (Figure 5F,G). That is, the friction force between regorafenib andVEGFR2 was enhanced. Furthermore, as the intensity increased, the VEGFR2-VEGFR2 friction force in the regorafenib solution decreased, meaning that the friction force between regorafenib and VEGFR2 increased (Figure 5F,G). The distribution of the measured friction force is shown in Figure 5F. Considering the correlation between friction force and adhesion force, the change in friction force between regorafenib and VEGFR2 under electric fields may also be attributable to the increase in internal energy and the change in the protein conformation of VEGFR2. The change in friction force between regorafenib and VEGFR2 also reflects the changes in hydrogen bonds, van der Waals forces, and hydrophobicity between VEGFR2 and regorafenib. The friction force between VEGFR2 molecules in the regorafenib solution under electric fields was positively correlated with adhesion force (Figure 5H). The induced conformational changes of VEGFR2 by the electric fields changed the friction interaction force between regorafenib and VEGFR2. This may be related to the increased internal energy and conformational transition rate of VEGFR2 due to the electric fields.

### 3.6. Electric Fields Affect the Constant-Load Friction Force Between Regorafenib and the Target Protein VEGFR2

**Figure 6:**
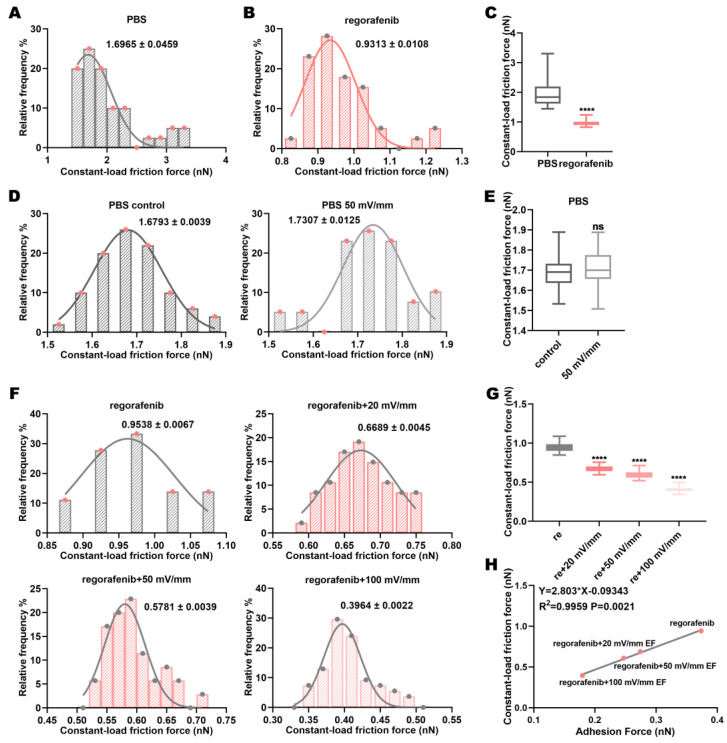
Electric fields reduce the constant-load friction force between VEGFR2-VEGFR2 molecules in regorafenib solution. (**A**) Distribution of VEGFR2-VEGFR2 constant-load friction force in PBS and (**B**) regorafenib solutions; (**C**) comparison of these forces. (**D**) Distribution of VEGFR2-VEGFR2 constant-load friction force in PBS solution alone and in PBS solution combined with electric fields; (**E**) comparison of these forces. (**F**) Distribution of VEGFR2-VEGFR2 constant-load friction force in regorafenib solution alone and in regorafenib solution combined with electric fields; (**G**) comparison of these forces. (**H**) The correlation between the constant-load friction force and adhesion force between VEGFR2 molecules after treatment with regorafenib alone or in combination with electric fields. The values in the constant–load friction force distribution graph represent the mean ± SD values. “ns” indicates “not statistically significant”; and **** indicates *p* < 0.0001.

The application of loading force can induce the movement of the protein [44,45,46]. The loading force applied via AFM acts directly on the protein [47]. In order to explore the influence of loading force on the protein–protein interaction in drug solution, we applied a loading force of 20 pN to the AFM probe to detect the constant-load friction force of VEGFR2-VEGFR2 in regorafenib solution under different electric field intensities. The results showed that the constant-load friction force of VEGFR2-VEGFR2 in regorafenib solution was significantly lower than that in the control group (PBS solution) (Figure 6A–C). In addition, the results showed that electric fields cannot affect the constant-load friction force of VEGFR2-VEGFR2 in PBS solution (Figure 6D,E). The constant-load friction force was significantly higher than the friction force, which also proved that the applied mechanical force can enhance the protein–protein interaction (Figure 5A–C and Figure 6A–C). Moreover, compared with regorafenib solution alone, the constant-load friction force of VEGFR2-VEGFR2 in regorafenib solution decreased under electric fields (Figure 6F,G). That is, the constant-load friction force of regorafenib–VEGFR2 was enhanced. With the increase in intensity, the constant-load friction force of VEGFR2-VEGFR2 in regorafenib solution decreased (Figure 6F,G). This revealed that the constant-load friction force of regorafenib–VEGFR2 increased. This was consistent with the change trend of the friction and adhesion forces, and it also proved the combination of regorafenib and VEGFR2 (Figure 6H and Figure S2D). The measured friction force distribution under constant load is shown in Figure 6F.

### 3.7. The Enhanced Inhibition of HCC by Regorafenib Under Electric Fields Is Related to the Force Between Regorafenib and VEGFR2

**Figure 7:**
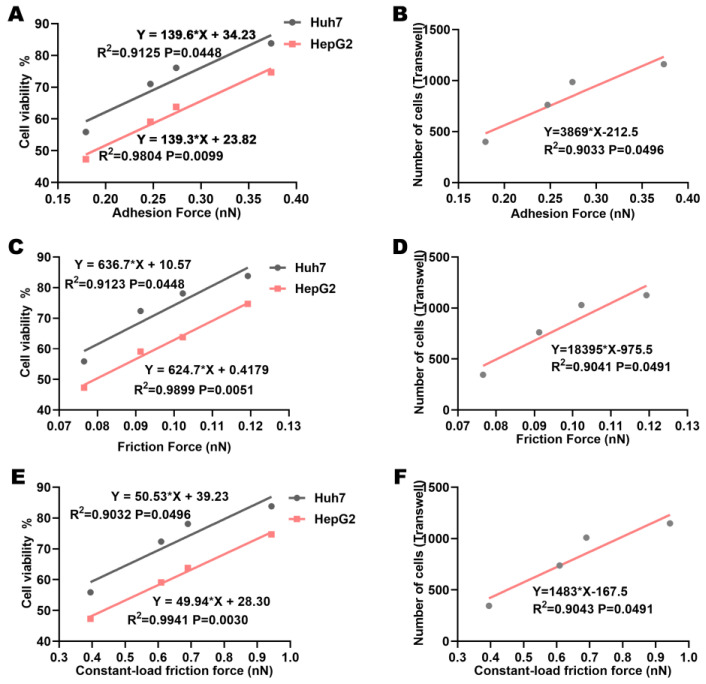
The enhancement of regorafenib’s inhibitory effect by electric fields may be related to the increased adhesion and friction forces between regorafenib and VEGFR2. Correlation of VEGFR2-VEGFR2 adhesion, friction, and constant-load friction forces with (**A**,**C**,**E**) cell viability and (**B**,**D**,**F**) cell migration.

Based on the above results, we inferred that the application of electric fields could promote binding and interaction between regorafenib and its target protein VEGFR2. This led us to wonder whether the enhanced inhibitory effect of regorafenib on HCC cells by electric fields was related to the increased interaction force between regorafenib and the target protein VEGFR2. It is worth noting that the trend of the adhesion and friction forces between VEGFR2 molecules under different electric field intensities was consistent with the change trend of cell viability and migration ability. The adhesion and friction forces between VEGFR2 molecules were significantly positively correlated with cell viability and migration ability (Figure 7A–D). In other words, the adhesion force (normal interaction force) and friction force (transverse interaction force) between regorafenib and VEGFR2 were negatively correlated with cell viability and migration ability. Different electric field intensities may enhance the inhibitory effect of regorafenib on HCC cell lines by enhancing the normal interaction and transverse interaction between regorafenib and VEGFR2, including the proliferation, invasion, and metastasis of HCC cell lines. The constant-load friction force of VEGFR2-VEGFR2 complexes was also significantly positively correlated with cell viability and migration ability (Figure 7E,F). This was consistent with the correlation of friction force with cell viability and migration ability. The constant-load friction force between regorafenib and VEGFR2 was also affected by electric fields.

### 3.8. The Morphology of VEGFR2 Changes Under Electric Fields

**Figure 8:**
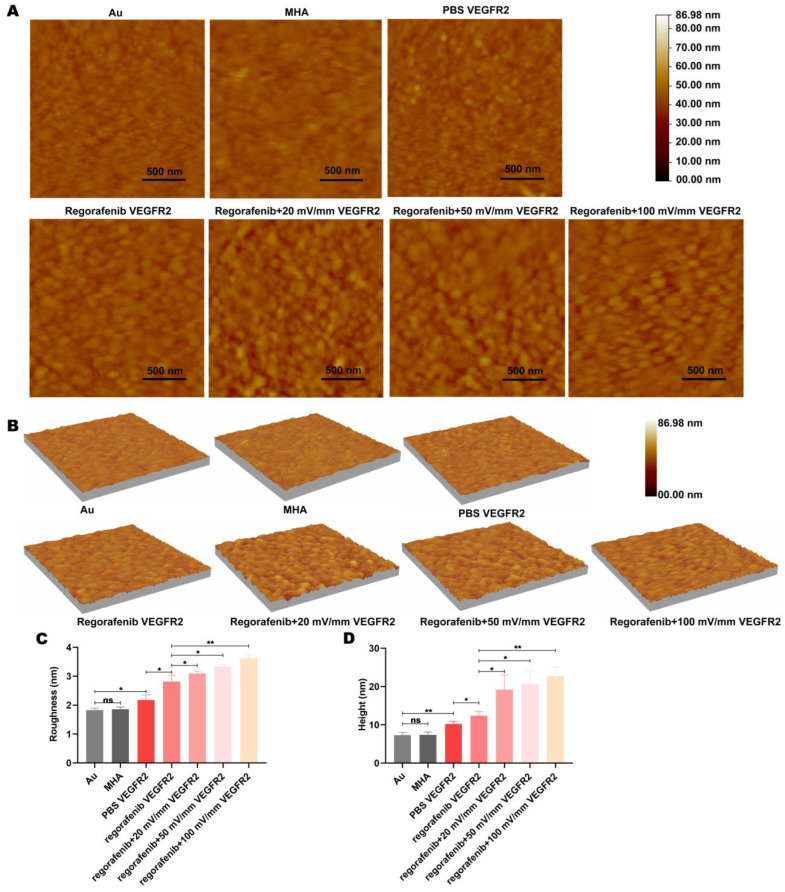
Electric fields cause a change in the surface morphology of the VEGFR2 molecular layer in regorafenib solution. (**A**) The 2D and (**B**) 3D morphology, (**C**) mean height, and (**D**) average roughness of Au, MHA molecular layers, VEGFR2 molecular layers in PBS solution, VEGFR2 molecular layers in regorafenib solution alone, and VEGFR2 molecular layers in regorafenib solution combined with electric fields. “ns” indicates “not statistically significant”; * indicates *p* < 0.05; and ** indicates *p* < 0.01.

AFM can detect the surface morphology of macromolecules [24,25,48]. A change in protein morphology can reflect a change in protein properties [19]. The surface morphology of the gold sheet and VEGFR2 protein molecular layer was detected by AFM. Compared with the gold sheet, the height and roughness of the VEGFR2 protein layer were significantly increased (Figure 8A–D). Protein particles of VEGFR2 could be observed from the topography (Figure 8A,B). Compared with the gold sheet, the contact angle of VEGFR2 protein molecular layers in water decreased (Supplementary Figure S2E,F). The above results demonstrated that the VEGFR2 protein molecular layer was successfully prepared.

The morphology of the VEGFR2 protein molecular layer in 5 μM regorafenib solution was examined. The protein particle size of VEGFR2 in regorafenib solution was larger than in PBS solution (Figure 8A,B). This may be due to the binding of regorafenib to VEGFR2, leading to the hydrophilicity of VEGFR2. Moreover, the height and roughness of the VEGFR2 molecular layer in regorafenib solution were increased (Figure 8C,D). These indicated that the protein morphology changed after regorafenib bound to VEGFR2.

The above results described the changes in VEGFR2-VEGFR2 adhesion and friction forces in regorafenib solution under electric fields. Changes in adhesion and friction forces reflect the alteration in nonbonded interactions such as hydrogen bonding, van der Waals forces, and hydrophobicity, which are closely related to protein conformation. Therefore, we further investigated whether the conformational changes in VEGFR2 and the enhanced interaction force between regorafenib and VEGFR2 under electric fields would be reflected in the morphological changes in the VEGFR2 protein layer. It was observed that VEGFR2 protein particle size in regorafenib solution increased under electric fields (Figure 8A,B). The height and roughness of the VEGFR2 protein layer in regorafenib solution were significantly improved under electric fields (Figure 8C,D). Moreover, compared with treatment using regorafenib solution alone, the contact angle of the VEGFR2 protein layer under treatment with different electric field intensities and regorafenib solution decreased (Supplementary Figure S2G). This may be due to increased binding between VEGFR2 and regorafenib as a result of the electric fields, resulting in an increase in the hydrophilicity of the VEGFR2–regorafenib complex. Increased hydrophilicity led to increased hydration, resulting in an increase in the size and height of the VEGFR2–regorafenib complex. Therefore, the surface morphology changes in the VEGFR2–regorafenib complex under electric fields can reflect the binding of regorafenib to VEGFR2. Furthermore, the changes in the conformation and properties of the VEGFR2–regorafenib complex under the action of electric fields may be the cause of changes in the adhesion and friction forces between VEGFR2 molecules.

## 4. Discussion

In this study, we explored whether the anti-tumor effect of TKI could be enhanced by electric fields and verified whether this was related to enhanced drug–protein interaction. We found that electric fields enhanced the inhibitory effect of regorafenib on the activity, invasion, and metastasis of HCC cell lines. Moreover, with an increase in electric field intensity, the inhibitory effect of regorafenib on the activity, invasion, and metastasis of HCC cell lines was enhanced.

The adhesion, friction, and constant-load friction forces between VEGFR2 molecules in regorafenib solution were reduced under electric fields. The adhesion force and friction force represent the normal interaction force and transverse interaction force between molecules, respectively. We used the protein–protein interaction force in the drug solution to reflect the drug–protein interaction force. On that basis, we inferred that the interaction between regorafenib and VEGFR2 was enhanced under electric fields (Supplementary Figure S2F). The interaction between regorafenib and VEGFR2 hindered the normal interaction and transverse interaction between VEGFR2 molecules. Moreover, with the increase in electric field intensity, the adhesion force and friction force between VEGFR2 molecules decreased, meaning that the adhesion force and friction force between regorafenib and VEGFR2 increased. The reduction in normal interaction force was manifested as specific and non-specific forces. In essence, this indicates that the hydrogen bonds, van der Waals forces, and hydrophobic properties between regorafenib and VEGFR2 changed. The VEGFR2-VEGFR2 adhesion and friction forces at different electric field intensities were positively correlated with the activity and migration ability of HCC cell lines. In other words, the adhesion and friction forces between regorafenib and VEGFR2 at different electric field intensities were correlated with the activity and migration ability of HCC cell lines. Therefore, we concluded that the electric field enhanced the inhibition of regorafenib in HCC cells by promoting binding and interaction between regorafenib and the target protein VEGFR2. This may be attributed to the conformational changes in the VEGFR2 protein induced by the electric fields. The energy provided by electric fields may cause an increase in the internal energy of the VEGFR2 protein and maintain it in a more stable conformation that is more easily bound to VEGFR2. The changes in drug–protein adhesion and friction forces essentially reflected the changes in protein properties and conformation. The increase in VEGFR2 protein height, particle size, and hydrophilicity under electric fields also reflected the change in protein properties and conformation, as well as the binding between regorafenib and VEGFR2.

At present, most studies aiming to improve the anti-tumor effect of regorafenib and other targeted therapeutic drugs rely on the synergistic combination of multiple drugs to achieve superposition of efficacy [3,49]. An applied electric field is a physical method to enhance the anti-tumor effect of targeted therapy drugs without the addition of other drugs. Although we only explored the effect of an electric field on the antitumor effects of regorafenib, these results suggest that applying an electric field may enhance the antitumor effects of other targeted therapeutic drugs, such as sorafenib, lenvatinib, and cabozantinib. Our study discovered the influence of electric fields on the binding between the drug and the target protein, which indicates the possibility of employing an electric field as an aid in molecular targeted therapy to enhance drug efficacy. It is possible that the binding of any ligand to its receptor could be modulated by an electric field. Naturally, our work is merely a preliminary validation. The specific electric field parameters utilized in clinical tumor therapy and their safety and effectiveness still require further exploration. Additionally, the correlation identified in this study between drug efficacy and the strength of interaction with the drug’s target protein offers a novel perspective for the development of targeted drugs. The detection of the interaction force between drugs and target proteins might be a potential method for validating drug effectiveness.

In conclusion, we have verified that the anti-tumor effect of regorafenib can be enhanced by applying electric fields, which act by enhancing the interaction between regorafenib and VEGFR2. The interaction force between regorafenib and VEGFR2 under electric fields was elucidated. Enhancing the anti-tumor effect of molecular targeted therapy drugs by changing features of the external environment such as the electric field is a feasible approach.

## Data Availability

All datasets generated or analyzed during this study are included in this article and are available from the corresponding author upon reasonable request.

## Supplementary Materials

The following supporting information can be downloaded at: https://www.mdpi.com/article/10.3390/biom15010042/s1, Figure S1: Electric stimulation device. Figure S2: (A) Cell viability of Huh7 cells and HepG2 cells under electric fields for 24 h. (B) Western blot original images of Figure 1G. (C) Schematic diagram of AFM force detection. (D) The correlation between the constant load friction force of VEGFR2-VEGFR2 and the friction force of VEGFR2-VEGFR2 after treatment with regorafenib alone or in combination with electric fields. (E) Schematic diagram of the contact angle. (F) The contact angle of Au, VEGFR2 molecular layers in PBS solution, VEGFR2 molecular layers in regorafenib solution alone and in regorafenib solution combining with electric fields. (G) Schematic diagram of enhanced normal and transverse interaction between regorafenib and VEGFR2 under electric field.
