# Supplementary material for: Effect of Electric Fields on the Mechanical Mechanism of Regorafenib–VEGFR2 Interaction to Enhance Inhibition of Hepatocellular Carcinoma
Source: Biomolecules. 2025 Jan 1;15(1):42. doi: 10.3390/biom15010042 (PMC11764289; doi:10.3390/biom15010042)
Supplement: Supplementary file 1 [file biomolecules-15-00042-s001.zip › biomolecules-3323890-supplementary.pdf]

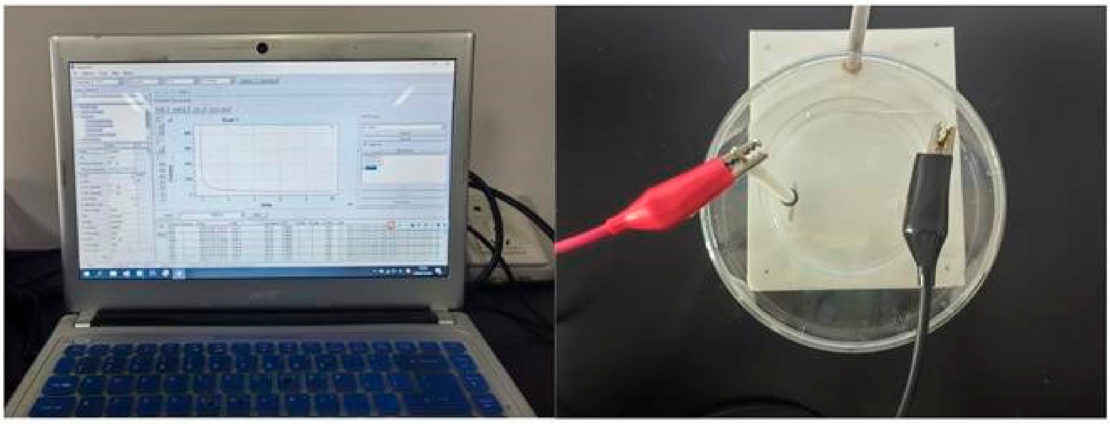

Figure S1. Electric stimulation device.

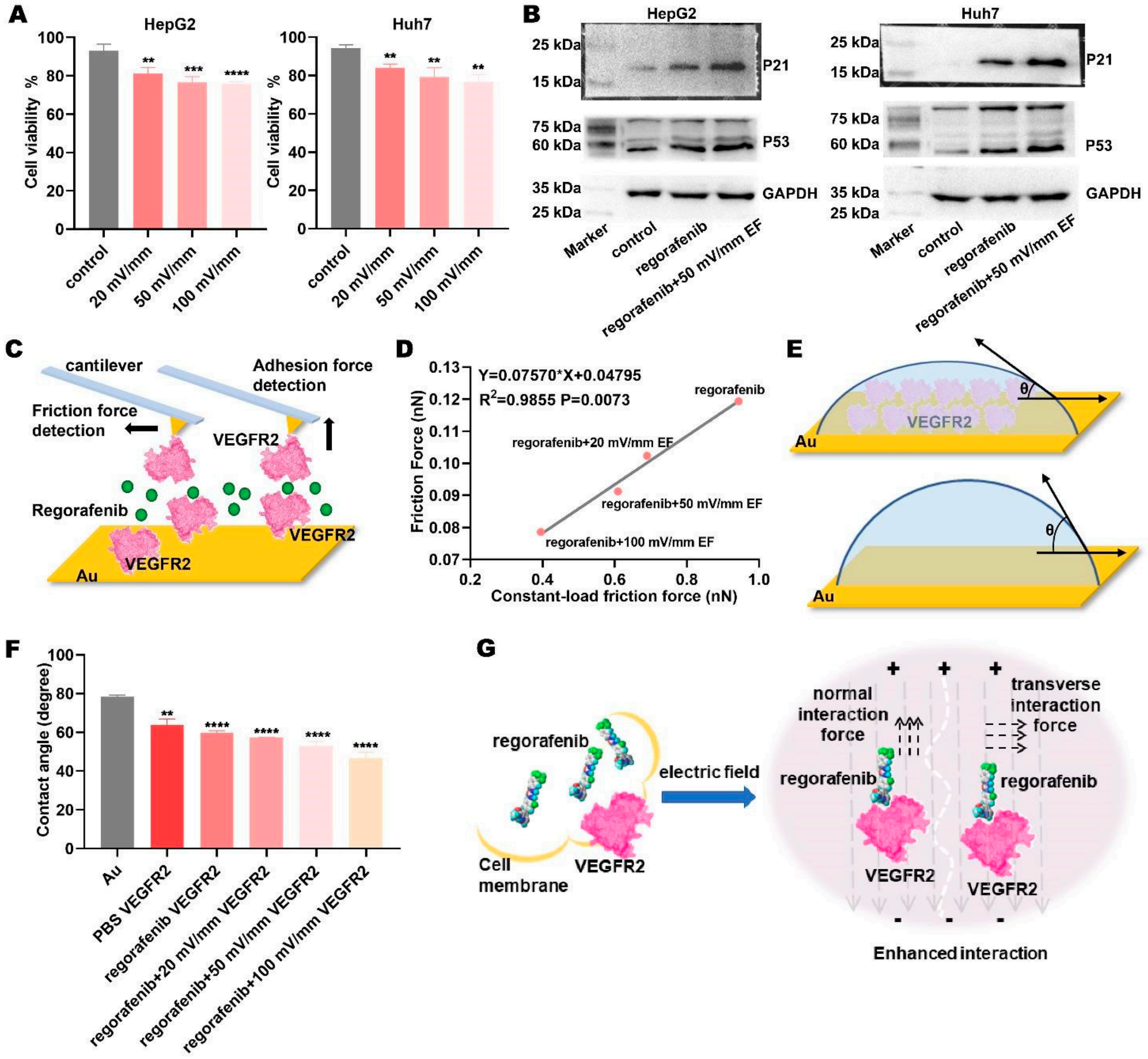

Figure S2. (**A**) Cell viability of Huh7 cells and HepG2 cells under electric fields for 24h. (**B**) Western blot original images of Figure 1G. (**C**) Schematic diagram of AFM force detection. (**D**) The correlation between the constant load friction force of VEGFR2-VEGFR2 and the friction force of VEGFR2-VEGFR2 after treatment with regorafenib alone or in combination with electric fields. (**E**) Schematic diagram of the contact angle. (**F**) The contact angle of Au, VEGFR2 molecular layers in PBS solution, VEGFR2 molecular layers in regorafenib solution alone and in regorafenib solution combining with electric fields. (**G**) Schematic diagram of enhanced normal and transverse interaction between regorafenib and VEGFR2 under electric field.
